# Seroprevalencia de *Brucella canis* en perros de un refugio para animales de compañía en Bogotá, Colombia

**DOI:** 10.7705/biomedica.5409

**Published:** 2021-06-25

**Authors:** Astrid-Jullieth Laverde, Daniela Restrepo-Botero, Diego Hernández-Pulido, José Luis Rodríguez-Bautista, Isabel-Sofía Sandoval

**Affiliations:** 1 Facultad de Medicina Veterinaria, Fundación Universitaria Agraria de Colombia, Bogotá, D.C., Colombia Fundación Universitaria Agraria de Colombia BogotáD.C Colombia; 2 Programa de Pós-Graduatjáo em Ciencias Veterinárias, Universidade Federal Rural do Rio de Janeiro, Seropédica, Rio de Janeiro, Brasil Universidade Federal Rural do Rio de Janeiro Universidade Federal Rural do Rio de Janeiro Rio de Janeiro Brazil

**Keywords:** brucelosis, perros, zoonosis, cromatografía de afinidad, salud pública, Brucellosis, dogs, zoonoses, chromatography, affinity, public health

## Abstract

**Introducción.:**

El riesgo de infección con *Brucella canis* en humanos y perros aumenta con la exposición constante a perros portadores asintomáticos. En Colombia hay evidencia de infección con *B. canis* en personas que conviven con perros. Una preocupación adicional en Bogotá es la falta de información actualizada sobre la prevalencia de la infección en perros destinados a programas de adopción.

**Objetivo.:**

Establecer la seroprevalencia de la infección por *B. canis* en perros de un refugio para animales de compañía destinados a la adopción en Bogotá.

**Materiales y métodos.:**

Se hizo un estudio descriptivo de corte transversal en un refugio para animales de Bogotá. Se detectaron anticuerpos contra *B. canis* en el suero de 51 perros (28 hembras y 23 machos) mediante una prueba inmunocromatográfica de flujo lateral. Asimismo, los individuos positivos se analizaron con PCR para la detección del ADN de *Brucella* spp.

**Resultado.:**

La seroprevalencia de *B. canis* fue del 1,96 % (1/51). El perro seropositivo correspondió a una hembra asintomática de tres años de edad en la cual no se detectó ADN bacteriano en sangre mediante la PCR.

**Conclusiones.:**

La seroprevalencia representada por un solo perro con IgG anti-*B. canis* puede considerarse un riesgo potencial para las poblaciones de perros y humanos, ya que podría tratarse de un animal con infección persistente capaz de diseminar la bacteria.

## Abstract (en)

**Introduction:**

The risk of *Brucella canis* infection in humans and dogs has increased due to the permanent exposure to asymptomatic carrier dogs. In Colombia, there is evidence of *B. canis* infection in humans living with dogs. In the case of Bogotá, an additional concern is the lack of updated information related to the prevalence of the infection in dogs.

**Objective:**

To determine the seroprevalence of infection by *B. canis* in dogs intended for adoption programs in Bogotá.

**Materials and methods:**

By means of a descriptive cross-sectional study carried out in a dog shelter in Bogotá, anti-*B. canis* IgG antibodies were detected in the serum from 51 dogs (28 females and 23 males) using a lateral-flow immunochromatographic test. Additionally, seropositive animals were analyzed with PCR to detect *Brucella* spp DNA.

**Results:**

*Brucella canis* seroprevalence was 1.96% (1/51). The seropositive dog was an asymptomatic three-year-old she-dog in which no bacteria DNA was detected in the blood through PCR.

**Conclusions:**

The seroprevalence determined in this study represented by a single dog with anti-*B. canis* IgG can be considered a potential risk both for canine and human populations since this single dog could have a persistent infection capable of spreading the bacteria.

La bacteria *Brucella canis* es considerada el principal agente causal de la brucelosis canina ^1^^-^^3^, aunque otras especies del género, como *B. abortus, B. suis y B. melitensis*, pueden infectar de forma transitoria a los perros ^4^. El cuadro clínico en el perro es variable y se encuentran desde casos asintomáticos ^1^^,^^5^^,^^6^, hasta abortos, orquitis, epididimitis, prostatitis ^2^^,^^7^, discoespondilitis ^8^, endoftalmitis ^9^ o linfadenomegalia ^10^. La transmisión a los animales vulnerables suele darse por medio del coito, los aerosoles o por contacto directo de mucosas o piel lesionada con material contaminado con el agente patógeno ^2^^,^^11^.

Se sabe que los fluidos vaginales posteriores a un aborto y el semen de animales infectados podrían constituir algunas de las fuentes más importantes de contagio para otros perros y para las personas, dada la elevada concentración de bacterias presentes en estas secreciones ^11^^,^^12^. Sin embargo, como *B. canis* puede persistir en secreciones vaginales no asociadas con el período de celo (estro) y en órganos diferentes a los reproductivos, el agente patógeno puede también diseminarse de manera intermitente y en bajas concentraciones en orina, saliva y secreciones nasales, por lo que la castración de hembras y machos no elimina totalmente el riesgo de contagio ^2^^,^^11^^-^^14^.

El tratamiento antibiótico para la brucelosis canina no siempre es efectivo para erradicar la bacteria, por lo que pueden presentarse picos recurrentes de bacteriemia después de finalizado el tratamiento ^15^^,^^16^, lo cual podría aumentar el riesgo de diseminación bacteriana a individuos vulnerables.

El contacto directo con semen, secreciones, membranas fetales y cachorros abortados de hembras infectadas, sigue siendo la principal ruta de transmisión al ser humano ^17^^,^^18^. La infección también puede ocurrir por medio de fómites y se sabe que la bacteria puede sobrevivir por largos períodos en el suelo y el agua contaminados ^11^^,^^19^^,^^20^.

Es muy probable que la infección por *B. canis* en los seres humanos se encuentre subdiagnosticada debido a la sintomatología inespecífica ^3^^,^^4^^,^^13^, y a que la bacteria no se puede identificar mediante las pruebas serológicas empleadas de rutina para el diagnóstico de otras especies del género ^13^. Aunque todavía se consideran poco frecuentes, un número creciente de estudios en la última década ha documentado infecciones con *B. canis* como causa de endocarditis, peritonitis y otros cuadros clínicos ^4^. En Colombia, la bacteria se aisló por primera vez en el 2009 en la sangre de una mujer asintomática propietaria de un criadero, cuyos perros presentaban problemas reproductivos asociados con la infección por *B. canis*^21^. Posteriormente, Sánchez-Jiménez, *et al*., informaron dos casos no publicados de médicos veterinarios seropositivos y con sintomatología sugestiva de brucelosis ^22^.

Desde el punto de vista zoonótico, quizá lo más relevante de esta bacteria es el riesgo ocupacional que representa para veterinarios, criadores de perros y empleados de refugios o perreras ^18^. No obstante, es poco el énfasis en el riesgo de transmisión por perros destinados a la adopción. Aunque *B. canis* se considera de bajo potencial zoonótico en comparación con otras especies del mismo género, se ha demostrado que el contacto estrecho con perros infectados aumenta el riesgo de contagio ^13^^,^^17^^,^^23^, especialmente en adultos mayores, niños e individuos inmunocomprometidos ^2^^,^^24^. La zoonosis por *B. canis* parece ser un problema emergente en residentes de barrios urbanos marginales y de bajo nivel socioeconómico, en los que sean numerosos los perros callejeros ^4^.

La seroprevalencia de *B. canis* en algunos albergues de caninos en Colombia varía entre 0 % en Envigado ^25^ y Bucaramanga ^26^, y 6,78 % en Medellín ^27^. No hay publicaciones recientes de estudios en refugios de Bogotá. En el ámbito mundial, los resultados también varían, pues se han encontrado prevalencias que oscilan entre 0,4 y 20,9 % en perros de refugios o domésticos ^10^^,^^11^^,^^24^^,^^28^^-^^32^.

En el diagnóstico serológico y como prueba de tamización, puede emplearse la detección cualitativa de anticuerpos IgG anti-*B. canis* mediante inmunocromatografía rápida (Anigen™), con 93,0 % de sensibilidad y 100,0 % de especificidad ^33^; posteriormente, es posible recurrir a pruebas complementarias como la de aglutinación rápida en placa con 2β-mercaptoetanol (PARP-2ME) ^19^.

El cultivo de sangre se considera la prueba de referencia, pero tiene poca sensibilidad y los resultados se demoran, por lo que la reacción en cadena de la polimerasa (PCR) puede considerarse una prueba de ayuda diagnóstica complementaria en el seguimiento o vigilancia de la enfermedad, pues detecta pequeñas cantidades del ADN bacteriano. La amplificación de un fragmento del gen *bp*26, que codifica para la proteína periplasmática e inmunodominante BP26 de *Brucella* spp. ^34^, con una sensibilidad de 92,6 % y una especificidad de 90,0 % frente al hemocultivo, puede ser la prueba de elección o complementaria para el diagnóstico de la infección por *B. canis*^35^.

Teniendo en cuenta la escasa información epidemiológica sobre esta zoonosis, el objetivo de este estudio transversal fue determinar la seroprevalencia de *B. canis* en perros de un refugio para animales de compañía ubicado en Bogotá.

## Materiales y métodos

Se hizo un estudio descriptivo de corte transversal en un refugio de animales ubicado en Bogotá, que funciona como albergue transitorio de perros abandonados en la zona urbana, donde permanecen hasta su adopción, muerte o eutanasia.

### Tamaño de la muestra

Para el cálculo del tamaño de la muestra (n) en una población de 145 perros, se empleó el módulo “Tamaño de muestra: detectar enfermedad (muestreo aleatorio y diagnóstico perfecto”, de la plataforma *Working in Epidemiology* (WinEpi) (http://www.winepi.net/f101.php). La prevalencia mínima esperada se fijó en 5,0 % con un nivel de confianza del 95,0 % y se obtuvo un número de 51 individuos seleccionados mediante muestreo aleatorio por conveniencia.

### Toma de muestras

Después de obtener el consentimiento informado de la propietaria del refugio, se tomaron muestras de sangre por punción de las venas yugular o cefálica de los animales, en tubos al vacío sin anticoagulante (Vacutainer system™); las muestras se rotularon con la respectiva identificación del animal y se refrigeraron hasta su envío al laboratorio. Los sueros se obtuvieron por centrifugación a 1.008*g* durante 5 minutos y, posteriormente, se congelaron a -20 °C en tubos de polipropileno de 1,5 ml (Eppendorf Tubes™) hasta el momento de su procesamiento.

### Diagnóstico serológico

Los anticuerpos (IgG) contra *B. canis* se detectaron usando un inmunoensayo de cromatografía de flujo lateral (Bionote, Inc., kit de inmunocromatografía Prueba Rápida Anigen™, Gyeonggi-do, Corea) siguiendo las especificaciones del fabricante. Brevemente, se adicionaron 10 μl de suero al pozo de la muestra, a continuación, se agregaron 2 gotas (aproximadamente 80 μl) de solución tampón diluyente y se interpretaron después de 20 minutos de incubación a temperatura ambiente. La presencia de dos bandas de color (banda *T* y banda *C*) en la ventana de resultados, se interpretó como un resultado positivo de anticuerpos contra *B. canis*^33^.

Las muestras positivas por inmunocromatografía se remitieron a un laboratorio externo de referencia para la detección de IgG mediante la prueba de aglutinación rápida en placa con 2β-mercaptoetanol (PARP-2ME) ^19^. El procedimiento empleado por el laboratorio fue el siguiente: se mezclaron a partes iguales el 2-mercaptoetanol y el suero problema, y la mezcla se expuso al antígeno de la cepa M de *B. canis*. La aglutinación indicó que el suero era positivo para IgG contra *B. canis*.

### Diagnóstico molecular

Las muestras de los animales seropositivos se analizaron por PCR para *Brucella* spp. en ADN extraído a partir de sangre completa previamente recolectada en tubos BD Vacutainer™ con citrato de sodio y los oligonucleótidos BMEI0535F (5'-GCGCATTCTTCGGTTATGAA-3') y BMEI10536R (5'-CGCAGGCGAAAACAGCTATAA-3'), reportados por Sánchez-Jiménez, *et al*., los cuales amplifican un fragmento de 451 pb del gen *bp*26 de *Brucella*^35^.

Las condiciones usadas fueron las mismas empleadas por Sánchez- Jiménez, *et al*., y Olivera, *et al*. ^35^^,^^36^: la solución de reacción contenía 3 μl de MgCl_2_ (3 mM, Fermentas, Foster City, CA, USA), 2,5 μl de solución tampón Tris- HCl 10 mM; tritón 100X y KCl con pH de 8,8 (Fermentas, Foster City, CA, USA), 0,5 μl de dNTP (10 mM), 3 μl de plantilla de ADN (1 ng/μl), 0,625 μl de cada oligonucleótido (0,25 μM), 0,2 μl de ADN de polimerasa Taq (5 UI/μl, Fermentas Taq DNA polimerasa recombinante, Foster City, CA, USA) y se empleó agua destilada para completar un volumen final de reacción de 25 μl ^35^.

La amplificación se hizo en un termociclador PTC 100™ (Perkin-Elmer Inc., San José, CA, USA) en un total de 25 ciclos de desnaturalización durante un minuto a 95 °C, alineamiento de los oligonucleótidos a 64 °C durante 45 segundos, y extensión de la cadena a 72 °C durante tres minutos. Finalizados los 25 ciclos, se realizó una extensión final a 72 °C durante seis minutos ^36^. Los productos de la PCR se visualizaron en una electroforesis en gel de agarosa al 1 % con 0,5 μg/ml de bromuro de etidio y la ayuda de un transiluminador ultravioleta (Transilluminator Mini Benchtop Model M-10E™, UVP, Upland, CA, USA). Se utilizó el marcador de peso molecular de 50 pb (50 a 1.000 pb), Generuler™ (Fermentas Inc., Burlington, Canadá) ^35^.

Como control positivo de la prueba, se utilizó el ADN extraído de la cepa de *B. canis* menos mucoide (M-) empleada en la preparación del antígeno para pruebas serológicas. Como control negativo, se emplearon todos los reactivos de la PCR, excepto el ADN ^35^.

### Análisis de los datos

Se elaboró una base de datos en formato Excel™, en la que se registró la información relevante de los perros evaluados: sexo, edad y estado reproductivo (enteros o castrados, sometidos a orquiectomía u ovariohisterectomía). Mediante el inmunoensayo de cromatografía de flujo lateral, se estableció la seroprevalencia para *B. canis* y se comparó por sexo, grupo etario (grupo I: ≤1 año; grupo II: >1 año y <6 años, y grupo III: ≥6 años) y estado reproductivo (castrados o enteros) en el grupo de estudio.

### Consideraciones éticas

El proyecto fue aprobado por el Comité de Ética y Bioética de la Fundación Universitaria Agraria de Colombia, después de verificar que los protocolos propuestos se ajustaran a las normas éticas para toma de muestras clínicas en animales, de conformidad con lo establecido en el Artículo 88 y el literal e) del Artículo 87 de la Resolución 8430 de 1993 (Normas científicas, técnicas y administrativas para la investigación en salud), y los artículos 15 y 16, Capítulo I, Título II de la Ley 576 de 2000 (Código de ética para el ejercicio profesional de la medicina veterinaria, la medicina veterinaria y zootecnia y zootecnia).

## Resultados

Se analizaron los sueros de 51 perros procedentes de un refugio para animales de compañía, de los cuales 28 eran hembras y 23 machos, 44 eran castrados y siete eran enteros. La edad de los animales fluctuaba entre los 4 meses y los 15 años (cuadro 1).

**Cuadro 1:** Prevalencia de anticuerpos contra *Brucella canis* según, sexo, edad y estado reproductivo de 51 perros de un refugio de mascotas en Bogotá

	Total (n)	Seronegativos n (%)	Seropositivos n (%)
Sexo			
Macho	23	23 (100)	0 (0)
Hembra	28	27 (96,4)	1 (3,6)
Edad (años)			
Grupo I (≤1)	10	10 (100)	0 (0)
Grupo II (>1 y <6)	23	22 (95,7)	1 (4,3)
Grupo III (≥6)	18	18 (100)	0 (0)
Estado reproductivo			
Castrado	44	43 (97,7)	1 (2,3)
Entero	7	7 (100)	0 (0)
Total	51	50 (98,04)	1 (1,96)

**Figura 1:**
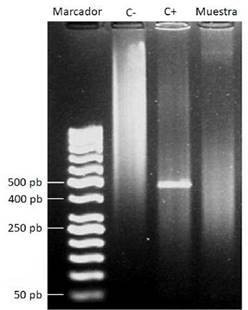
Electroforesis en gel de agarosa al 1 % de la PCR de un fragmento del gen *bp26* de *Brucella* spp. en muestra de sangre completa del perro seropositivo para IgG anti-*B. canis*. Marcador: marcador de peso molecular (Generuler 50 bp DNA Ladder™); C-: control negativo (agua); C+: control positivo. ADN de una cepa (M-) de *B. canis*, amplicón de 451 pb, aproximadamente; muestra: muestra de sangre negativa

La seroprevalencia para *B. canis* por inmunocromatografía fue de 1,96. Por otro lado, ninguno de los machos analizados fue seropositivo, en tanto que una hembra (3,6 % del total de hembras) fue seropositiva, tanto por inmunocromatografía como por PARP-2ME, aunque sin evidencia de ADN bacteriano en la sangre en la PCR (figura 1). En cuanto al estado reproductivo, la hembra con anticuerpos anti-*B. canis* estaba esterilizada, lo que se presenta en el 2,3 % de los animales bajo esta condición. Con respecto a la edad, la serorreacción fue de 0,0, 4,3 y 0,0 % para los grupos I, II y III, respectivamente (cuadro 1).

## Discusión

La seroprevalencia de *B. canis* en el inmunoensayo de cromatografía de flujo lateral en los perros del refugio fue del 1,96 %, correspondiente a una hembra de tres años sin signos clínicos de brucelosis. Esta seroprevalencia difiere de la encontrada en otros estudios en animales de albergues en diferentes regiones de Colombia, y es mayor a la observada en los estudios desarrollados en las ciudades de Envigado ^25^ y Bucaramanga ^26^, donde no se encontraron animales seropositivos mediante inmunocromatografía. Por el contrario, la seroprevalencia encontrada en este trabajo fue menor que la reportada en los estudios realizados en animales de albergue en Villavicencio y Medellín, en los que se determinaron valores de 9,3 % ^37^ y 6,78 % ^27^, respectivamente, mediante la prueba de aglutinación rápida en placa con 2p-mercaptoetanol (PARP-2ME).

Contrario a esto, la seroprevalencia encontrada en Bogotá fue mayor que la obtenida por Pardo, *et al*., en un estudio mixto con perros callejeros y domésticos, la cual fue de 1,49 % empleando la prueba PARP-2ME ^38^.

Por otro lado, cabe resaltar que la seroprevalencia hallada en este estudio fue menor que la reportada en trabajos previos con perros domésticos en tres ciudades colombianas. En Medellín, fue del 2,76 % según la inmunocromatografía ^39^ y del 6,6 % con la PARP-2ME ^40^ en perros de asentamientos urbanos de bajo perfil socioeconómico (comunas), y del 11,0 %, en perros de criaderos o de clínicas veterinarias según la prueba de PARP-2ME ^23^. En Montería, la seroprevalencia encontrada con inmunocromatografía en hembras de entornos urbanos fue de 11,0 % ^41^. También, vale la pena resaltar que la seroprevalencia hallada es mucho menor que la observada por Castillo, *et al*., (citado por Giraldo, *et al*.), la cual correspondió al 20,3 % en una clínica veterinaria universitaria de Bogotá en el 2002 ^23^.

Por otro lado, en el ámbito mundial los reportes de prevalencia de *B. canis* con base en serología o cultivo también se caracterizan por el alto índice de variabilidad, ya que se encuentran valores entre 0,40 y 20,9 % ^10^^,^^11^^,^^28^^-^^32^.

El amplio rango de prevalencias reportadas en Colombia y otros países podría atribuirse a múltiples factores, entre ellos, el método de muestreo, las características de la población estudiada ^2^, y la sensibilidad y especificidad de las pruebas de diagnóstico empleadas ^42^.

Es importante tener en cuenta que la seroconversión contra *B. canis* se inicia con el incremento de IgM hacia la tercera o cuarta semana, lo que se puede extender hasta la semana 12 después de la exposición ^13^^,^^43^^,^^44^. Entonces, pueden esperarse falsos negativos cuando las pruebas serológicas se hacen antes de la seroconversión o, en infecciones crónicas, cuando los títulos de anticuerpos circulantes se encuentran por debajo de los niveles de detección de las pruebas serológicas ^13^^,^^44^^-^^46^. Por consiguiente, dado que la reacción de los anticuerpos a una infección reciente o leve varía según las situaciones que se presenten después de la exposición, la técnica serológica empleada podría no detectar infecciones recientes o infecciones crónicas que se estuviesen presentando en la población de estudio.

Además de la dinámica de la respuesta humoral a la infección por *B. canis*, es necesario señalar que las características inherentes a toda prueba diagnóstica inciden directamente en el análisis de los resultados. En este caso, la poca probabilidad de encontrar falsos positivos (especificidad del 100 %) contrasta con la posibilidad de obtener falsos negativos (sensibilidad del 93 %) ^33^. Por consiguiente, debe considerarse que la seroprevalencia en la población de estudio puede ser superior a la encontrada.

No se pudo demostrar la presencia de ADN de *B. canis* con la prueba de PCR en el individuo seropositivo, pese a ser este un método más sensible para determinar la infección que el hemocultivo y la serología ^46^. La bacteriemia por *B. canis* tiende a desarrollarse hacia la segunda a cuarta semana de la infección y es de carácter intermitente ^45^. Durante la bacteriemia, el agente patógeno coloniza los tejidos diana como órganos reproductivos, hígado, bazo y médula ósea ^15^^,^^37^^,^^38^^,^^46^^-^^48^, o puede localizarse en los ganglios linfáticos ^49^^,^^50^. Por ello, es posible obtener PCR positivas sin seroconversión al inicio de la infección ^13^, o PCR negativas en animales seropositivos que se encuentren en la fase crónica de la infección con localización de la bacteria en órganos blanco ^13^^,^^46^^-^^48^, como posiblemente sucedió en este caso.

No se encontró IgG en otros perros en estrecho contacto con la hembra infectada. Además de la sensibilidad de la técnica diagnóstica, puede haber otros factores asociados con la presencia de animales seronegativos en la población de estudio. Aspectos como la castración del 86,3 % de los animales y la consiguiente ausencia de prácticas reproductivas, pudieron contribuir a minimizar el riesgo de contagio ^51^^,^^52^.

El impacto de esta infección animal en la salud de los seres humanos no ha sido completamente caracterizado porque gran parte de la información proviene de reportes de casos o informes de brotes ^11^^,^^17^^,^^53^^,^^55^,y, además, porque las pruebas serológicas disponibles en el mercado detectan especies de las cepas lisas de *Brucella* y no anticuerpos contra *B. canis*^56^, lo que ha conducido, finalmente, a que existan pocos estudios de prevalencia. Aunque desde el punto de vista ocupacional se considera que los cuidadores de los perros en los refugios o criaderos, los médicos veterinarios y el personal de laboratorio son los principales grupos de riesgo ^18^, con la evidencia científica también se destaca como factor de riesgo potencial el tener perros como animales de compañía ^11^, especialmente para los niños ^55^^,^^57^, las personas inmunocomprometidas ^53^^,^^54^, las de edad avanzada ^58^ o las mujeres gestantes ^59^.

El aumento descontrolado de la población canina causa un impacto negativo en la salud pública ^60^. Los perros callejeros representan un factor de riesgo de infección para las personas y para otros animales ^32^^,^^60^^,^^61^. Las enfermedades en perros con tutores responsables son relativamente más fáciles de controlar, en comparación con la población callejera, puesto que esta deambula por la calle y tiene más probabilidades de entrar en contacto con otros perros infectados y luego convertirse en potenciales diseminadores del agente patógeno ^32^. Por lo tanto, las estrategias de diagnóstico no deberían excluir las poblaciones de perros callejeros, con el fin de determinar la prevalencia e implementar las medidas pertinentes de control de *B. canis* en estos grupos.

La presencia de perros asintomáticos potencialmente adoptables podría constituir un gran riesgo para la población humana y animal, lo que debe alertar al personal sanitario sobre la importancia de hacer tamizaciones periódicas para detectar la infección en los dos grupos poblacionales.

Los resultados del presente estudio aportan información actualizada de la seroprevalencia de *B. canis* en un refugio de perros en Bogotá y pueden ser la base para futuras investigaciones de la enfermedad, tanto en perros callejeros como en animales de compañía.
